# Novel curcumin- and emodin-related compounds identified by *in silico *2D/3D conformer screening induce apoptosis in tumor cells

**DOI:** 10.1186/1471-2407-5-97

**Published:** 2005-08-05

**Authors:** Melanie Füllbeck, Xiaohua Huang, Renate Dumdey, Cornelius Frommel, Wolfgang Dubiel, Robert Preissner

**Affiliations:** 1 Institute of Biochemistry, Charité, Universitätsmedizin Berlin, Monbijoustr. 2, 10117 Berlin, Germany; 2 Division of Molecular Biology, Department of Surgery, Charité, Universitätsmedizin Berlin, Monbijoustr. 2, 10117 Berlin, Germany

## Abstract

**Background:**

Inhibition of the COP9 signalosome (CSN) associated kinases CK2 and PKD by curcumin causes stabilization of the tumor suppressor p53. It has been shown that curcumin induces tumor cell death and apoptosis. Curcumin and emodin block the CSN-directed c-Jun signaling pathway, which results in diminished c-Jun steady state levels in HeLa cells. The aim of this work was to search for new CSN kinase inhibitors analogue to curcumin and emodin by means of an *in silico *screening method.

**Methods:**

Here we present a novel method to identify efficient inhibitors of CSN-associated kinases. Using curcumin and emodin as lead structures an *in silico *screening with our in-house database containing more than 10^6 ^structures was carried out. Thirty-five compounds were identified and further evaluated by the Lipinski's rule-of-five. Two groups of compounds can be clearly discriminated according to their structures: the curcumin-group and the emodin-group. The compounds were evaluated in *in vitro *kinase assays and in cell culture experiments.

**Results:**

The data revealed 3 compounds of the curcumin-group (e.g. piceatannol) and 4 of the emodin-group (e.g. anthrachinone) as potent inhibitors of CSN-associated kinases. Identified agents increased p53 levels and induced apoptosis in tumor cells as determined by annexin V-FITC binding, DNA fragmentation and caspase activity assays.

**Conclusion:**

Our data demonstrate that the new *in silico *screening method is highly efficient for identifying potential anti-tumor drugs.

## Background

The COP9 signalosome (CSN), a conserved multimeric protein complex, functions at the interface between signal transduction and ubiquitin (Ub)-dependent proteolysis [1]. Because of associated enzymes, the CSN possesses kinase acitivity. Two of the associated kinases are the protein kinase CK2 (CK2) and the protein kinase D (PKD) [2]. More than 200 proteins are known to be phosphorylated by the CK2, which is located nearly everywhere in the cell. The PKD is a serine/threonine kinase localized at either the plasma membrane or the cytosol of lymphocytes [3] and is associated with very diverse cellular functions, including Golgi organization, plasma membrane directed transport, metastasis, immune response, apoptosis and cell proliferation [4]. It is assumed that the CSN is a platform that brings together the kinases and appropriate substrates [5]. Transcriptional regulators such as p53 and c-Jun are phosphorylated by the CSN kinases [6,7]. The phosphorylation of p53 at Thr155 results in Ub-dependent degradation of the tumor suppressor [6]. In contrast, the CSN-directed phosphorylation of c-Jun leads to the stabilization of the transcription factor towards the Ub/26S proteasome system [8]. Cellular functions such as regulation of transcription, DNA repair, cell cycle regulation, senescence and apoptosis are modulated by p53 as well as c-Jun. Defects most frequently observed during tumorigenesis are mutations in the p53 gene [9]. It is well known that wild type p53 provides a critical brake in tumor development [10]. In contrast, as a component of the activator protein-1 the onco-protein c-Jun is mostly a positive regulator of cell proliferation and involved in oncogenic transformation (for review see [11]). Hence, the intracellular concentrations of p53 and c-Jun are decisive for tumor development. Therefore, in tumor therapy it is of great interest to control the stability of p53 and c-Jun in tumor cells. One strategy might be the inhibition of CSN-associated kinases, CK2 and PKD. It has been demonstrated before that blocking CSN-mediated phosphorylation causes an increase of p53 [6] and a decrease of c-Jun [12], very useful effects for anti-tumor drugs. Curcumin has been identified as an inhibitor of CSN-associated kinases [13], which is already in phase I clinical trials for evaluations concerning the prevention of colon, breast, lung and prostate cancer [14]. Former investigations showed that curcumin is a potent inhibitor of angiogenesis [15] and of the recombinant kinases CK2, PKD and the purified CSN complex from erythrocytes [2,13]. In addition, a natural product called emodin is also known as an inhibitor of the CK2 (PDB-Code: 1F0Q), PKD and the CSN complex [2].

In this study we developed an *in silico *screening to identify novel, more effective inhibitors of CSN-associated kinases by using our in-house database (more than 10^6 ^compounds). Curcumin and emodin served as lead structures in the screenings. Using a 3D superposition algorithm [16] the lead structures were compared with every compound of the database. For better coverage of the compounds and to assure their flexibility during usage of the algorithm a total of ~50 conformers were computed for every compound of the database. Compounds identified from the *in silico *screening were evaluated in kinase assays and cell culture experiments. With the new screening strategy potential new drugs for tumor therapy were identified, which stabilized endogenous p53 and induced apoptosis in tumor cells.

## Methods

### *In silico *screening

#### Three dimensional (3D) similarity search

Lead structures (curcumin and emodin) and compounds in the database were prepared for the 3D search, which is based on structural similarities. As a first step the centers of mass of each compound were determined and superimposed. The plane and the straight line of minimal quadratic distance to all atoms were computed to determine the least and largest (orthogonal) expansion. One structure was rotated such that the major directions coincide. In a further step the normalization of the atomic set was used to identify pairs of corresponding atoms. The root mean square distance (rmsd) was calculated for the related atomic pairs. An improvement of this value was obtained by removing or adding atoms to this superposition [16].

#### Two dimensional (2D) similarity search

Another possibility to find new inhibitors of CSN-associated kinases was the 2D search, which is based on chemical similarities. The presence or absence of common functional groups such as alcohols or ring systems such as pyrimidins was investigated. Each substructure element was assigned to one bit of a Boolean array. To calculate 2D similarities between two structures the Tanimoto-coefficient was estimated. Bits set in both structures (BitsAB) and bits, which were only set in one structure (BitsA and BitsB) were taken into consideration. The value varies between 0 (different molecules) and 1 (equal molecules): [17].

In further steps each compound was analyzed for its possible application as a drug. First we investigated the absorption and permeability using the Lipinski's rule-of-five, which implies that molecules should contain less than 10 H-bond-acceptors and less than 5 H-bond-donors. The calculated logP-value (describes the lipophilic properties) should be less than 5 and the molecular weight should be less than 500 g/mol [18]. Any compound violating more than one rule is not a promising candidate for a drug.

Based on the similar property principle one can predict toxic effects from the molecular structure. We used two quantitative structure toxicity relationship (QSTR) models to analyze the compounds for their toxicity. Using the software TOPKAT^® ^DS MedChem Explorer (Discovery Studio, Accelrys Inc., [19]) from Accelrys Inc., which comprises the QSTR models, the toxicological data were obtained [20].

### Kinase assay

Kinase activity was determined with [γ-^32^P]ATP (ICN) in a buffer containing 30 mM Tris, pH 7.6, 10 mM KCl, 0.5 mM DTT. Full-length c-Jun was used as substrate. Recombinant CK2 (2α2β) and recombinant PKD were obtained from Calbiochem and Upstate, respectively. The CSN was isolated from human erythrocytes as outlined before [7]. The protein kinase inhibitors emodin and resveratrol were purchased from Calbiochem, curcumin and piceatannol were from Sigma. BTB00363 (2-Pyridinecarboxylic acid, [(3,5-dichloro-2-hydroxyphenyl)methylene]hydrazide), SEW04213 (6-fluoro-3,4-dihydro-2H-pyrano [2,3-b]quinolin-5-amine), BTB14431 (9,10-dihydroxy-1,4-dihydroanthracene-1,4-dione), JFD02836 (3-methoxy-10-methyl-9,10-dihydro-9-acridinone) and JFD03665 (10-(hydroxymethylene)phenanthren-9(10H)-one) were obtained from Maybridge. Approximately 1 μg of full-length c-Jun was incubated with inhibitors at different concentrations (0, 20, 50, 100 and 200 μM) and 10 μCi [γ-^32^P]ATP for 60 min at 37°C. Reactions were stopped by adding 3 μl of 4 × SDS-sample buffer (Roth). The samples were separated by SDS-PAGE in a 12.5% gel, stained with Coomassie, dried and exposed to X-ray film. Subsequently the data were evaluated by densitometry. IC50 values were calculated assuming Michaelis-Menten kinetics.

### Cell culture

HeLa cells were cultured in RPMI 1640 medium containing 10% (v/v) fetal calf serum, 2 mM glutamine (Life Technologies, Inc.), penicillin (100 U/ml) and streptomycin (100 μg/ml) in a humidified 5% CO_2 _atmosphere. For inhibitor treatment, 5–7 × 10^6 ^HeLa cells were incubated with curcumin, resveratrol, piceatannol, BTB00363, emodin, SEW04213, BTB14431, JFD02836 or JFD03665 in 0.25% dimethyl sulfoxide (DMSO) for 4 h at indicated concentrations. Control cells were treated with 0.25% DMSO. After inhibitor treatment, cells were harvested and lysed using ice-cold mono-detergent lysis buffer (50 mM Tris pH 8.0, 1% Triton-X-100, 1 mM EDTA, 150 mM NaCl, 1 μg/ml aprotinin, 1 μg/μl PMSF). HeLa cell lysate proteins were separated by SDS-PAGE in a 12.5% gel, blotted to nitrocellulose and probed with anti-c-Jun antibody (Calbiochem). For testing the intracellular concentration of p53 by Western blot analysis, B8 cells (murine fibroblasts) were cultured using Iscoves's MEM medium (Biochrom) with 125 μg/ml G418 (Gibco BRL). Cells (5 × 10^6 ^B8 cells/well) were incubated with resveratrol, piceatannol, emodin, BTB14431 or JFD02836 for 4 h at indicated concentrations. Control cells were treated with 0.25% DMSO. After inhibitor treatment, cell lysates were produced and analyzed as described for HeLa cells. Proteins were probed with an anti-p53 antibody (IC Chemikalien). All Western blots were developed using the ECL technique (Amersham).

### Caspase-3/7 activity assay

To determine apoptosis, B8 cells (10^4 ^cells/well) were incubated with indicated inhibitors for 4 hours. After incubation, the caspase-3/7 reagent, containing a DEVD-peptide (Promega) was added as recommended by the manufacturer (Promega). The fluorescence of each well was measured at an excitation wavelength of 485 nm and an emission wavelength of 530 nm.

### Cell viability assay

Cell viability was assessed by the MTT [3-(4,5-dimethylthiazol-2-yl)-2,5-diphenyltetrazolium bromide] assay (Sigma-Aldrich), which is based on the ability of a mitochondrial dehydrogenase from viable cells to oxidize the tetrazolium rings of the pale yellow MTT and to form a dark blue formazan crystals, which are largely impermeable to cell membranes and, therefore, accumulate within healthy cells. The number of surviving cells is directly proportional to the concentration of the formazan produced.

Cells were incubated with the indicated inhibitors at different concentrations (20 or 50 μM) for 24 h. Then a solution of MTT in phosphate-buffered saline (PBS) was added to each well to a final concentration of 0.5 μg/μl. After 4 h incubation dark blue formazan was solubilized with 100 μl DMSO. Absorbance was measured at 590 nm using an ELISA reader.

### Annexin-V-FITC/propidium iodide (PI) double staining

Annexin V binds to phosphatidyl serine externalized to the outer leaflet of the plasma membrane bilayer during initial stages of apoptosis. To measure cell staining by annexin V the substance was labeled with FITC (fluorescein isothiocyanate). Simultaneously the cells were stained with PI. By the double staining the test discriminates between intact (FITC^-^/PI^-^), apoptotic (FITC^+^/PI^-^) and necrotic cells (FITC^+^/PI^+^) [21]. First, HeLa cells were incubated with the indicated inhibitors at different concentrations (20 or 50 μM) for 24 h. After harvesting and washing with PBS cells were resuspended in 100 μl annexin V binding buffer (containing 10 mM HEPES/NaOH, pH 7.4, 140 mM NaCl, 2.5 mM CaCl_2_). Subsequently cells were incubated with 5 μl of FITC-conjugated annexin V (ApoTarget) for 20 min at room temperature. After annexin-V-FITC staining, 400 μl of annexin V binding buffer containing PI (2.5 μg/ml) were added and the cells were analyzed by flow cytometry within 1 hour after staining.

### DNA-fragmentation

Cells were seeded at a density of 10^5 ^cells/ml and treated with different concentrations (20 and 50 μM) of the indicated emodin- and curcumin-like compounds. After 24 h the cells were collected, washed with PBS at 4°C and fixed in PBS/2% (vol/vol) formaldehyde on ice for 30 min. After fixation cells were incubated with ethanol/PBS (2:1, vol/vol) for 15 min, pelleted and resuspended in PBS containing 40 μg/ml RNase A. After incubation for 30 min at 37°C cells were pelleted again and finally resuspended in PBS containing 50 μg/ml PI. The nuclear DNA fragmentation was then quantified by flow cytometric determination of hypodiploid DNA. Data were analyzed using the CELLQuest software. Data are given in percentage of hypoploidy (subG1), which reflects the number of apoptotic cells.

## Results

*In silico *screening plays an important role in drug-design [21]. The method used here was developed to identify potential new inhibitors of the CSN-associated kinases, CK2 and PKD. Based on the structures of the known inhibitors curcumin and emodin [2] a search in our in-house database containing approximately 10^6 ^compounds was performed. A 3D superposition algorithm was developed to compare structures of the known inhibitors (lead structures) and the database compounds. Using the 3D superposition algorithm the identification of structures, which do not exactly fit into the pattern of the lead structure (scaffold hoppers, 2D similarity <85%), can be realized. To carry out specific searches, many features such as the size of a molecule (Å), limit of rmsd, number of assigned atoms and number of similar atoms were compared. With this approach 35 compounds similar to the lead structures curcumin and emodin were identified and further analyzed.

As a first step we tested the behavior of the compounds concerning the Lipinski's Rule-of-five using Accord for Excel from Accelrys Inc. Our investigation showed that no compound broke more than one rule (data not shown). Further the toxicological effects of the compounds were tested. Two QSTR models were employed: Rat Oral LD50 (Lethal Dose) and NTP (National Toxicology Program) Rodent Carcinogenicity. All identified compounds were predicted to be harmless.

**Figure 1:**
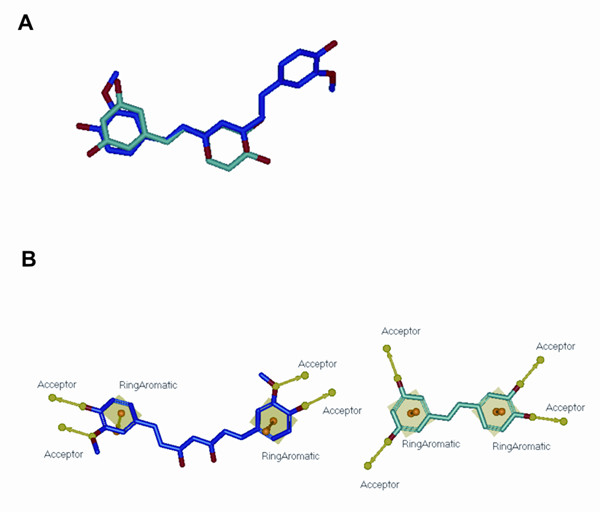
**3D superposition and 2D comparison of curcumin and piceatannol**. Comparison of curcumin and piceatannol structures by three-dimensional (3D) and two-dimensional (2D) superposition. *A*, The lead structure curcumin (blue) was aligned in a 3D superposition with the structure of piceatannol (green) obtained in the database. *B*, 2D similarities of curcumin and piceatannol are demonstrated. Both structures contain two aromatic rings and a large number of H-bond-acceptors. Using this information the Tanimoto-coefficient shown in Table 1 was calculated.

Based on the different lead structures and the calculated Tanimoto-coefficients the identified compounds were divided into two groups (see Table 1). The compounds of the first group were found by searching the database with curcumin and the compounds of the second group are related to emodin. Fig. 1A shows the superposition of the structures curcumin (blue) and piceatannol (green). In addition, the chemical properties of the two compounds are compared (Fig. 1B). Both structures contain two aromatic rings and a number of H-bond acceptors, which seem to be important for the inhibitory effect.

**Figure 2:**
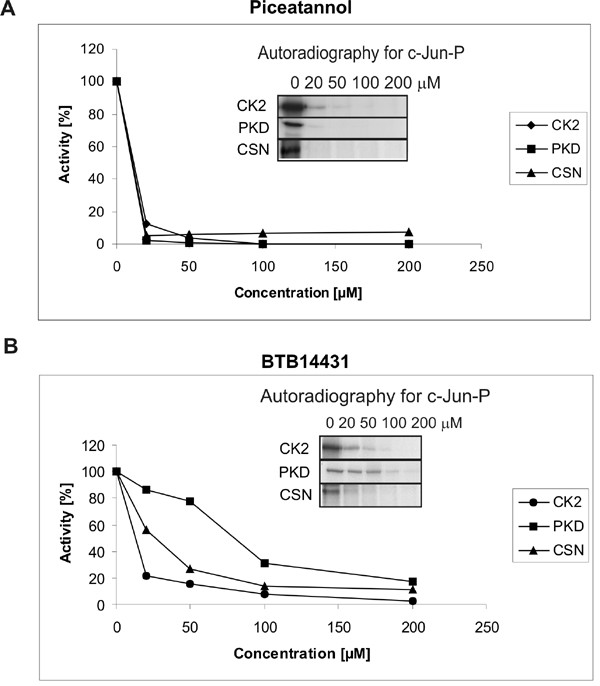
***In vitro *kinase assays with piceatannol and BTB14431**. Inhibition of CSN-associated kinases by curcumin- and emodin-derived compounds *in vitro*. *A*, Piceatannol, identified by searching the database with curcumin as a lead compound and *B*, BTB14431, a compound found by searching the database with emodin. Recombinant CK2 and PKD as well as purified CSN were used to phosphorylate c-Jun *in vitro*. The first lane (0) is without inhibitors. Piceatannol (A) and BTB14431 (B) were added in increasing concentrations (20, 50, 100 and 200 μM). The shown autoradiographs were evaluated by densitometry. The obtained data were plotted against increasing inhibitor concentrations and used to calculate IC50 values summarized in Table 2. The demonstrated results are representative for at least four independent experiments.

All 35 compounds selected from the database by the screenings described above were tested in kinase assays for their ability to inhibit CSN-associated kinases. In these assays recombinant CK2 or PKD as well as purified CSN were incubated in the presence of [γ-^32^P]ATP and 50 or 200 μM of the potential inhibitors (data not shown). The data showed that only 7 out of 35 identified compounds inhibited the CSN-associated kinases significantly in the chosen concentration range. Therefore 7 reagents, 3 compounds of the curcumin-group and 4 of the emodin-group, were used for further analysis. Next IC50 values of these 7 compounds were determined with recombinant CK2, PKD or with the purified CSN. Kinase assays were performed in the presence of different inhibitor concentrations. After incubation assays were analyzed by SDS-PAGE and autoradiography. Kinase activity (%) was estimated by densitometry. Fig. 2 demonstrates the results for piceatannol (curcumin-group) and BTB14431 (emodin-group). Values obtained by densitometry were plotted against inhibitor concentrations. Obtained curves were used to calculate IC50 values, which are summarized in Table 1 for all tested compounds and compared with the values for curcumin and emodin determined before [2]. Data show that curcumin-derived compounds seem to have a higher affinity for PKD whereas inhibitors from the emodin-group were more efficient in suppressing the CK2. In most cases IC50 values obtained with the purified CSN were lower than those with recombinant kinases (Table 1).

**Figure 3:**
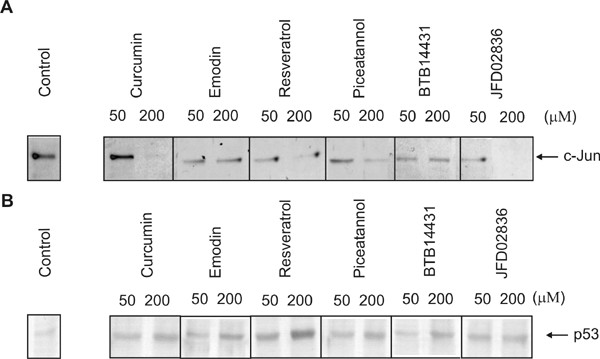
**Effect of curcumin- and emodin-derived inhibitors on the stability of c-Jun and p53**. Curcumin- and emodin-derived inhibitors of CSN-associated kinases affect the stability of c-Jun and p53 in HeLa or B8 cells. HeLa cells or B8 cells were incubated for 4 h with the inhibitors emodin, resveratrol, piceatannol, BTB14431 and JFD02836. The first lane is the control without inhibitor. Proteins of cell lysates were separated by SDS-PAGE and transferred to nitrocellulose. The Western blots analysis was probed with an anti-c-Jun or an anti-p53 antibody.

Because cell treatment with curcumin or emodin led to Ub- and proteasome-dependent degradation of c-Jun [2,12], we tested whether HeLa cells incubated with different concentrations of the new inhibitors possess decreased c-Jun levels. It can be seen in the Western blot analysis (Fig. 3A) that resveratrol as well as piceatannol (curcumin-group) and BTB14431 as well as JFD02836 (emodin-group) induced a significant reduction of endogenous c-Jun in a dose-dependent manner.

It has been shown before that curcumin stabilizes endogenous p53 toward the Ub system in HeLa and MCF7 cells [6]. These cell lines possess wild type p53 [22-26]. We were interested to see whether our new inhibitors of CSN-associated kinases also increase intracellular steady state p53 concentration. Mouse B8 fibroblasts, also expressing wild type p53 [27] were treated with inhibitors. Subsequently cell lysates were tested by Western blot analysis using an anti-p53 antibody. Data are shown in Fig. 3B. All tested compounds induced significant stabilization of endogenous p53 in B8 cells in a dose-dependent manner. Interestingly, resveratrol at 200 μM was most effective in stabilizing the tumor suppressor (Fig. 3B).

It has been shown that emodin, curcumin and resveratrol induce apoptosis through p53-dependent pathways [28,29]. Therefore we asked the question whether piceatannol, BTB14431, SEW04213 and JFD02836 also induce apoptosis. Several studies on compounds similar to curcumin have been published. The ability to induce apoptosis was measured in approximately 60 tumor cell lines and effects were obtained in nearly all cells examined [30]. Most studies demonstrate the execution of apoptosis by the oxidation of tetrazolium [30], the measurement of DNA-fragmentation as well as caspase activity and annexin-V-FITC/propidium iodide (PI) double staining [31]; methods also applied in our investigations.

**Figure 4:**
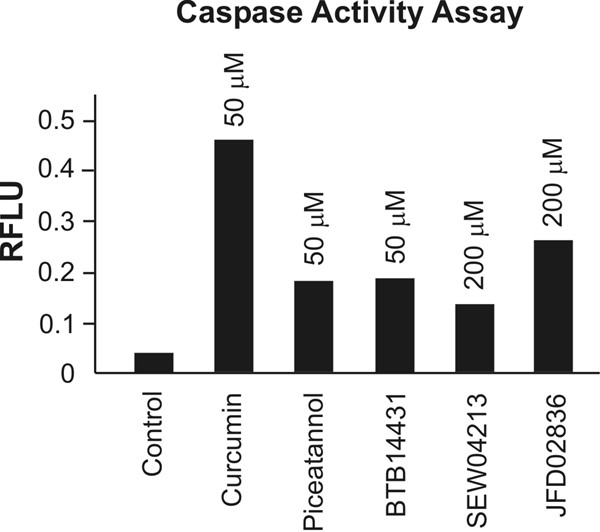
**Measurement of caspase activity after cell treatment with curcumin- and emodin-derived inhibitors**. Curcumin- and emodin-derived inhibitors of CSN-associated kinases induce apoptosis in B8 cells. Caspase-3/7 activities in mouse B8 fibroblasts were determined using a DEVD-peptide. Cells (10^4^/well) were treated with the inhibitors curcumin, piceatannol, BTB14431, SEW04213 and JFD02836 for 4 h at 37°C. After incubating cells with caspase-3/7 reagent for 3 h at room temperature the fluorescence (RFLU: relative fluorescent light units) was measured at an excitation wavelength of 485 nm and an emission wavelength of 530 nm.

First the caspase activity was measured in B8 fibroblasts. Data shown in Fig. 4 demonstrate that all inhibitors tested caused an increase of caspase activity. The most pronounced increase was obtained with 50 μM of curcumin. Piceatannol (50 μM) and BTB14431 (50 μM) elevated caspase activity by approximately 5-times, whereas significant effects with SEW04213 and JFD02836 were only obtained at compound concentrations of 200 μM.

**Figure 5:**
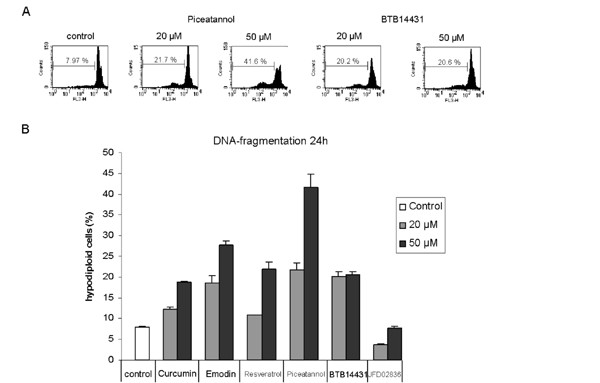
**Detection of DNA-fragmentation in HeLa cells after 24 h incubation with kinase inhibitors**. Dose-dependent DNA-fragmentation in HeLa cells after 24 h treatment with curcumin- and emodin-derived inhibitors. (A) Representative data for piceatannol (20 μM and 50 μM) and BTB14431 (20 μM and 50 μM) are shown. As a control the cells were incubated with DMSO (0.1%). Piceatannol and BTB14431 were added in two concentrations (20 μM and 50 μM). The marker indicates the percentage of subG1 cells (apoptotic cells). (B) The bar chart shows the percentage of hypodiploid cells (apoptotic cells) after treatment with the inhibitors curcumin, emodin, resveratrol, piceatannol, BTB14431 or JFD02836 at different concentrations (20 μM and 50 μM).

To corroborate these findings we investigated the DNA-fragmentation after treating HeLa cells with curcumin, emodin, resveratrol, piceatannol, BTB14431 and JFD02836 for 24 h. Except for JFD02836 in all experiments apoptosis was triggered (Fig. 5B) and the subG1 peak, which reflects the number of apoptotic cells increased in a dose-dependent manner (Fig. 5A).

**Figure 6:**
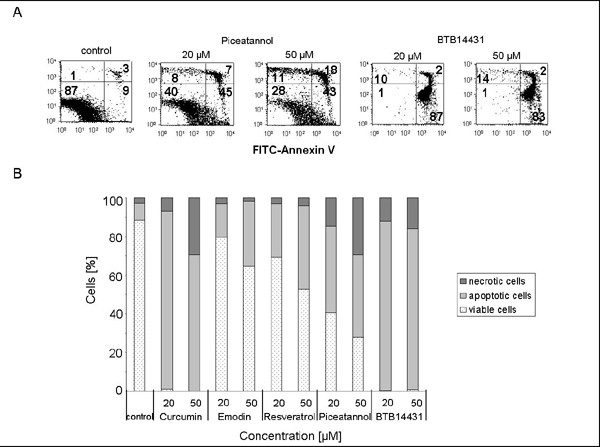
**Apoptosis or necrosis: Annexin-V-FITC /PI double staining of HeLa cells after treatment with CSN kinase inhibitors**. HeLa cells were cultured in medium containing 0.1% DMSO or the curcumin- and emodin-derived inhibitors at different concentrations (20 μM and 50 μM). After 24 h cell death was measured at the single-cell level by labeling cells with annexin-V-FITC and counterstaining with propidium iodide (PI). (A) Representative data for piceatannol and BTB14431 are shown. The numbers indicate the percentage of cells in each quadrant (lower left: FITC^-^/PI^-^, intact cells; lower right: FITC^+^/PI^-^, apoptotic cells; upper left: FITC^-^/PI^+^, necrotic cells; upper right: FITC^+^/PI^+^, late apoptotic or necrotic cells). (B) The bar chart describes the percentual distribution of necrotic, apoptotic and viable cells after treatment with curcumin, emodin, resveratrol, piceatannol or BTB14431.

In addition, by staining cells with annexin-V-FITC/PI cell death was quantified [32]. After 24 h of incubation a large number of apoptotic cells appeared in the experiments with the inhibitors curcumin, piceatannol as well as BTB14431 (Fig. 6B). Emodin and resveratrol produced less apoptotic cells. In experiments with high concentrations (50 μM) of curcumin and piceatannol (Fig. 6A) increased amounts of necrotic cells were observed.

**Figure 7:**
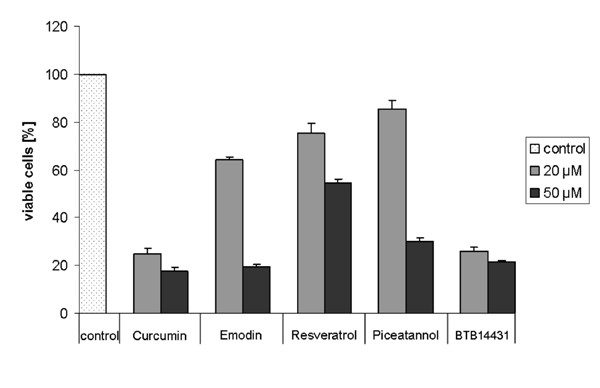
**MTT test for detecting the cell viability after treatment with kinase inhibitors**. The cells were treated with curcumin, emodin, resveratrol, piceatannol or BTB14431 for 24 h at different concentrations (20 μM and 50 μM). As a control the cells were cultured with 0.1% DMSO. The bar chart displays the amount of viable cells after treatment.

The viability of the cells was checked by the MTT test, which detects the percentage of irreversibly damaged cells after treatment with the indicated inhibitors. By adding curcumin and BTB14431 at a concentration of 20 μM the percentage of viable cells decreased to ~25%. A significant reduction of viable cells with emodin, resveratrol or piceatannol was only detected at high inhibitor concentrations (50 μM) (Fig. 7).

## Discussion and conclusion

### Virtual screening versus high-throughput screening

Recently we have shown that curcumin and emodin, inhibitors of CSN-associated kinases, induce Ub- and proteasome-dependent degradation of c-Jun in tumor cells [2,12]. Moreover, curcumin treatment causes stabilization of the tumor suppressor p53 towards the Ub system [6]. It has been demonstrated that curcumin- and emodin-induced increase of p53 results in massive tumor cell death due to p53-dependent apoptosis [28,29]. Therefore, at least in tumor cells with wild type p53 elevation of cellular p53 levels could be of high therapeutic effect. Both events, the reduction of c-Jun and the increase of p53 are important for tumor therapy and can be accomplished by inhibition of CSN-associated kinases.

Therefore a new method was developed to identify compounds which are effective blockers of CSN-associated kinases and can be potentially used in tumor therapy. In our *in silico *screening we referred to curcumin and emodin as lead structures and compared them with approximately 10^6 ^compounds of our in-house database regarding their structural properties (similar property principal). In contrast to high-throughput screening (HTS) our method allows to exclude a large number of compounds before experimental testing. HTS is often used and appropriate assay systems can evaluate more than 125,000 compounds a day. However, HTS is not without problems [33,34]. HTS experiments become more and more expensive and the handling of the large amount of data is very time consuming. In addition, it is difficult to exclude false-positive hits [33]. By our virtual screening method we identified 35 compounds that seemed to be promising candidates for CSN kinase inhibition. Out of the 35 structures found by *in silico *screening 7 compounds had an inhibitory effect on recombinant CK2 and PKD and on the kinase activity of the purified CSN complex. Thus, the hit rate of our virtual screening was 20%. In contrast, the hit rate of HTS is usually approximately 2% [35]. We have used additional methods such as the Lipinski rule-of five and the toxicological investigations for ranking the found substances. These methods, however, did not serve to exclude compounds. Summing up, the data demonstrate that our *in silico *screening is a reliable and efficient method to find new active molecules.

### The curcumin- and emodin-derived inhibitors of CSN-associated kinases

The present study includes only compounds which were identified using curcumin or emodin as lead structures. Our screening revealed 3 compounds of the curcumin-group and 4 compounds of the emodin-group, which showed inhibition of the kinases *in vitro *and in cell experiments. Interestingly, some of the identified compounds are more effective inhibitors than the lead structure. Therefore, *in silico *screening is a sensitive method for the identification of molecules with specific biological function. The two groups can be clearly divided by structural features. The different structures are most likely responsible for their different effects. Whereas members of the curcumin-group possess much better IC50 values with the PKD, the members of the emodin-group are better inhibitors of CK2. However, all compounds inhibit both CK2 and PKD relatively unspecifically. It has been shown before that emodin is a competitive CK2 inhibitor [36]. As demonstrated by crystal structure it binds into the ATP-binding pocket of the kinase [37]. Simulation on the basis of the emodin data revealed that curcumin also fits into the same ATP-binding pocket of the CK2 (data not shown). In addition, the effects of curcumin are reversible [12] as it would be expected of a competitive inhibitor. Therefore, we conclude that all identified compounds compete with ATP for the ATP-binding site of the kinases. Since the ATP-binding sites of CK2 and PKD are slightly different, members of the curcumin-group have another preference as compared to the emodin-group. On the other hand, ATP-binding sites are highly conserved among kinases. Therefore the identified kinase inhibitors are rather unspecific.

### Inhibitors of CSN-associated kinases are potential drugs for tumor therapy

Interestingly, many identified inhibitors of CSN-associated kinases are compounds of natural products such as curcumin, resveratrol, piceatannol, emodin and honokiol, which have been shown to inhibit angiogenesis and development of malignancies [12,15,38,39]. Here we demonstrate possible anti-tumor mechanisms of known and new substances. The inhibitors of CSN-associated kinases exhibit two important effects. As shown here they reduce c-Jun levels in tumor cells. In addition, it has been demonstrated that inhibitors of CSN-associated kinases cause an increase of intracellular p53, which can be explained by the stabilization of the tumor suppressor towards the Ub/26S proteasome system [6]. However, because our data were obtained by estimating steady state levels of p53, altered expression might also contribute to the increased protein concentration in the cells. Nonetheless, elevated steady state levels of p53 are accompanied with massive cell death caused by apoptosis as demonstrated here with B8 fibroblasts and HeLa cells. In addition, the viability of the cells decreased after treatment with the selected substances.

Based on our data we cannot exclude that the tested compounds exert their pro-apoptotic effects independently of the CSN and p53. For example, in addition to CSN-associated kinases curcumin inhibits NF-κB activation associated with the induction of apoptosis [40]. Moreover, although the exact role of c-Jun in apoptosis is not known, low c-Jun steady state levels as measured in our experiments also might contribute to the induction of the apoptotic program (for rev. see [11]). In any case, the identified inhibitors exert their effects by stimulating apoptosis in tumor cells, which is most beneficial for tumor therapy. Based on its anti-tumor potential the CSN kinase inhibitor curcumin is already in phase I clinical trials [14] and perhaps inhibitors identified here will follow.

## Competing interests

For compound BTB14431 a patent application is pending for assignee Charité.

## Authors' contributions

MF designed and carried out the study, interpreted the data and drafted the manuscript; RP, WD, CF conceived, coordinated, and designed the study, interpreted the data and drafted the manuscript; XH participated in the cell culture experiments; RD participated in the *in vitro *assays. All authors have read and approved the final manuscript.

## Pre-publication history

The pre-publication history for this paper can be accessed here:



## Figures and Tables

**Table 1:** Creation of two groups of potential inhibitors of CSN-associated kinases. The structures found in the database by 3D and 2D screening were divided into two groups (curcumin-group and emodin-group) depending on the lead structures curcumin or emodin. The analysis of the 2D structures abet the division into the two groups. The double line marks the threshold, which normally limits the hits (2D similarity <85%).

**Trade name**	**2D-structure**	**2D similarity (Tanimoto-coefficient)**
**Curcumin-group**		

Curcumin	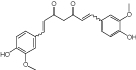	1.0
Resveratrol	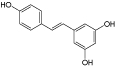	0.7
Piceatannol	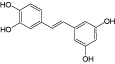	0.7
BTB00363	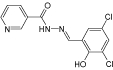	0.7

**Emodin-group**		

Emodin	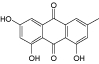	1.0
BTB 14431	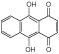	0.9
JFD02836	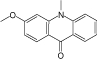	0.9
SEW 04213	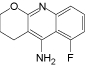	0.8
JFD03665	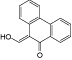	0.8

**Table 2:** Determination of IC50 values in an in vitro kinase assay. IC50 values (μM) for the inhibition of recombinant CK2, PKD and the purified CSN complex by the inhibitors of the curcumin-group, piceatannol as well as BTB00363, and of the emodin-group, BTB14431, JFD02836, SEW04213 and JFD03665 were determined. The two groups of inhibitors were created based on the different structures of the lead compounds (see Table 1). Kinase assays were performed as outlined in materials and methods. For comparison IC50 values of curcumin, resveratrol and emodin from earlier measurements [2] are shown.

**Inhibitor**	**IC50 CK2 (μM)**	**IC50 PKD (μM)**	**IC50 CSN (μM)**
**Curcumin-group**			

Curcurmin	11.8	4.1	2.6 *
Resveratrol	51.0	17.6	32.1 *
Piceatannol	2.5	0.5	1.7
BTB00363	332.7	117.7	188.6

**Emodin-group**			

Emodin	22.7	94.5	4.4 *
BTB14431	6.4	68.9	21.0
JFD02836	19.9	39.7	54.1
SEW04213	37.9	185.9	4.8
JFD03665	5.9	154.9	19.8

* [2]
